# In-silico and in-vitro studies to identify potential inhibitors of SARS-CoV-2 spike protein from Omani medicinal plants

**DOI:** 10.1016/j.heliyon.2024.e39649

**Published:** 2024-10-24

**Authors:** Nabras Al-Mahrami, Smitha Sunil Kumaran Nair, Adhra Al Mawali, Raja-Mohamed Beema Shafreen, Saeed Ullah, Sobia Ahsan Halim, Ahmed Al-Harrasi, Nallusamy Sivakumar

**Affiliations:** a National Genetic Center, Royal Hospital, Ministry of Health, Sultanate of Oman; b Department of Computing and Electronics Engineering, Middle East College, Sultanate of Oman; c Quality Assurance and Planning, German University of Technology (GUtech), Sultanate of Oman; d Department of Biotechnology, Dr Umayal Ramanathan College for Women, Karaikudi, India; e Natural and Medical Sciences Research Center, University of Nizwa, Sultanate of Oman; f Department of Biology, Sultan Qaboos University, Sultanate of Oman

**Keywords:** Aeyan Al Aqrada, Coronavirus, Corilagin, COVID-19, Drug discovery, Ethnomedicine, In-silico, In-vitro, Molecular docking, Molecular dynamics simulation, SARS-CoV-2 spike protein, Sultanate of Oman

## Abstract

In the quest for novel therapeutic agents against SARS-CoV-2, the proposed study explores the potential of traditional Omani medicinal plants, focusing on the efficacy of natural ligands against the virus's Spike protein. Among 437 identified medicinal plants across Oman, 47 species that are documented for their traditional use in treating respiratory infections, with 30 species' ligands available were chosen for analysis. Molecular docking was performed using Autodock Vina on these ligands, yielding 406 unique ligands post-duplication removal. The binding affinities of target-ligand complexes were precisely determined, ranking them by interaction strength. This process identified Corilagin, a phytochemical from the Acalypha indica plant (locally known as Aeyan Al Aqrada), as the most promising inhibitor. Subsequent analyses using GROMACS for molecular dynamics simulation confirmed its binding stability and interaction dynamics of the Corilagin-protein complex. The in-vitro studies further validated Corilagin's inhibitory effect on SARS-CoV-2, demonstrating a remarkable 92 % inhibition at 0.5 mM concentration. Dilution studies to ascertain the IC_50_ value revealed Corilagin's high potency at a micromolar level (IC_50_ = 2.15 ± 0.13 μM), underscoring its potential as a drug candidate for SARS-CoV-2 treatment. These findings highlight the significance of ethnomedicine and in-silico methodologies in drug discovery, offering promising directions for future antiviral research.

## 1 Introduction

The global acceptance of innovations, encompassing the utilization of traditional medicines is embraced by the World Health Organization (WHO) in its pursuit of identifying potential treatments for COVID-19 showcasing the importance of ethnomedicine-based treatments [1]. One such assistance is the WHO Global Centre established for Traditional Medicine (GCTM) in India in 2022 which serves as a hub of knowledge in the realm of traditional medicine, aligning with WHO's broader strategy in this field by prioritizing evidence, learning, data analytics, sustainability, equity, innovation, and technology to enhance the role of traditional medicine in global health and sustainable development, all while upholding the principle of respecting local heritage, resources, and rights [2].

Ethnomedicine is popular for its large storage of active chemicals and pharmacological characteristics with its ability to modulate immune system components. However, there is not yet a commercially available antiviral substance made from natural materials [3]. There has been an increase in the research focus on medicinal plants as potential candidates for an effective vaccine and antiviral medicine ever since the coronavirus illness 2019 (COVID-19) was declared to the world in December 2019. The immunological effects of medicinal herbs are advantageous since they serve as the first line of defense. There is a plethora of evidence showing the potential of ethnomedicine in the treatment of several disease conditions [4].

Despite the vaccines, the demand for novel therapies against coronaviruses persists, given the diverse therapeutic properties exhibited by plant-derived compounds. Leveraging the established safety and efficacy of these bioactive compounds in clinical studies for other infectious diseases, there is a heightened potential for their application against coronavirus too [5].

Bioinformatics approaches have significantly contributed to drug discovery by utilizing computational methods such as molecular docking and molecular dynamics simulations [6]. Molecular docking allows researchers to investigate the binding affinity of potential drug candidates with target proteins, unraveling important information about the strength and specificity of interactions. It has been used as a rapid computational screening method for vast libraries of chemicals [7,8]. There are several examples of commonly used molecular docking software, namely AutoDock Vina [9,10], Schrödinger's Glide [11] and SwissDock [12]. Adding Molecular dynamics simulations to this offering precise insight into offering precise insights into the stability and behavior of the drug-target complex over time, mimicking physiological settings [13]. GROMACS is one of the most popular and widely open-source used, it is optimized for high-performance computation, considering it ideal for the simulation of biomolecular systems such as nucleic acids, lipids, and protein [14]. The integration of molecular docking and molecular dynamics simulations provides a computational - in silico-pipeline that aids the investigation of a potential drug candidate by giving insight into the dynamic nature of molecular interactions. By incorporating in-vitro studies, the candidate drug can be experimentally validated in biological systems. This confirmation step ensures that chemical compounds exhibit the impact in real conditions, strengthening the overall findings and guiding further optimization. An example case of combining in-silico and in-vitro studies for drug discovery and progressing into the clinical trials phase is Molnupiravir (MK-4482/EIDD-2801) [15], used as an antiviral medicine that works by stopping the virus that causes COVID-19 from growing and spreading. Such discovery showed the robust application of computational pipelines to conduct more studies. Our study primarily focuses on testing traditional Omani medicinal plants' ligands against the SARS-CoV-2 spike protein (S) using in-silico and in-vitro methods.

## 2 Literature review

Synthetic drugs rarely associate with the root cause of diseases but treat the disease symptoms. Moreover, synthetic inhibitors may have side effects. On the other hand, ethnomedicine-based drugs have less or no toxicity and hence safer to use. However, due to the relatively limited understanding of COVID-19's pathophysiology, it is imperative to subject potential plant-based drug molecules to thorough in silico, in vitro, and in vivo analyses, ensuring they possess a robust pharmacokinetic profile before advancing to clinical trials. This approach addresses concerns regarding their efficacy and challenges associated with poor bioavailability [16].

Teka and Maryo (2023) evaluated the usage of plant species in Ethiopian traditional medicine for treating respiratory tract infections by compiling essential data to support future scientific inquiries, drawing from published articles and grey literature. The review covers aspects such as medicinal plant diversity, growth forms, plant parts used, preparation methods, sources, distribution, and commonly treated respiratory conditions. A total of 229 plant species utilized for respiratory disorders in Ethiopia were identified. The information gathered is a valuable resource for selecting plants for subsequent pharmacological, phytochemical, and toxicological studies, potentially leading to novel plant-derived therapeutic interventions [17].

A study conducted in Zimbabwe aimed to catalog plants that have been traditionally utilized in the country for more than 40 years to treat respiratory conditions and identified 58 families of medicinal plants with 160 species. Their assessment results in a total of 44 native and 12 exotic medicinal plant species as possible targets for additional toxicological analysis and clinical investigation into their capacity to treat COVID-19 (Nyagumbo et al., 2022) [18]. The review carried out by Ogunrinola et al. (2021) identified a list of medicinal plants that have been investigated in the past in Nigeria for their potential to treat provoked symptoms that appear in COVID-19 infection [19]. Given the multifaceted clinical presentation of COVID-19, especially in individuals with coexisting medical conditions, Oladele et al. (2020) advocate for a comprehensive approach involving combination therapy that entails the administration of two or more Nigeria-based medicinal plants, each possessing distinct therapeutic properties, to effectively target the various mediators of the disease [20]. Saliu et al. (2021) conducted in-silico pharmacokinetic screening and molecular docking analyses using 100 phytocompounds derived from fourteen Nigerian plants (Crinum jagus, Andrographis paniculata, Sage plants (Salvia officinalis L.), and Anacardium occidentale) as ligands, targeting nsp16 (PDB: 6YZ1). Initially, only 59 phytocompounds passed the drug-likeness analysis. Subsequently, docking analysis identified six phytocompounds (oxopowelline, andrographolide, deacetylbowdensine, 11, 12-dimethyl sageone, sageone, and quercetin) displaying strong binding affinity with nsp16 at its active site [21].

In Northern Peru, 91 plant species from 82 genera and 48 families were recorded and classified as herbal respiratory system medicines. As per the study, most herbal remedies for respiratory disorders were made from plant leaves (27.69 %), whereas the plant's entire composition (18.46 %), flowers (13.85 %), and stems (17.69 %) were used less frequently. The knowledge acquired about commonly used herbal treatments provides some ideas for potential targets for additional research to produce new pharmaceuticals [4]. A review conducted by Boozari and Hosseinzadehand (2020) identified the potential targets as S protein (emodin, baicalin) and viral enzymes replication such as PLpro (Cryptotanshinone), 3CLpro (Iguesterin), RdRp (Sotetsuflavone), and helicase (Silvestrol) which can be inhibited by natural agents [22].

The symptoms of COVID-19, particularly the challenging long COVID-19, have prompted researchers to explore natural products for health enhancement and symptom relief. Pranskuniene et al. (2022) conducted an archival investigation, focusing on safety guidelines, to uncover potential remedies for respiratory ailments in Lithuanian traditional medicine. Their findings revealed the usage of 60 medicinal plant species from 29 diverse families, with 28 out of these 60 species not featured in the European Medicines Agency (EMA) monographs, and only half of the included species aligning with the EMA's recommendations for respiratory system disorders. However, this served as an important source of ideas for further investigations of ethnomedicine-based pharmaceutical preparations for COVID-19 symptoms [23].

A study conducted by Mlozi (2022) encompassed a phytochemical assessment of medicinal plants employed against COVID-19 in Tanzania, involving the collection of plant materials from traditional practitioners and subsequent qualitative screening to ascertain the presence of secondary metabolites. These findings highlight the burgeoning interest in pharmaceutical research targeting phytochemical extracts from medicinal plants and aromatic herbs, intending to identify potential lead compounds for developing antiviral medications [24].

AYUSH-64, a poly-herbal formulation developed by the Ministry of AYUSH, Government of India, has demonstrated both safety and efficacy in treating infectious febrile conditions like malaria and influenza after extensive research including pharmacological, toxicological, and clinical studies. The components of AYUSH-64 exhibit potential in mitigating initial COVID-19 symptoms, suppressing the cytokine storm, inhibiting Angiotensin-Converting Enzyme II (ACE2), and reducing Reactive Oxygen Species (ROS), thus preventing the progression of COVID-19. Building upon the findings of clinical trials for influenza-like illnesses and molecular docking studies, AYUSH-64 has been repurposed and evaluated in several clinical trials as a standalone therapy or in conjunction with standard care for asymptomatic and mild to moderate COVID-19 cases, yielding promising results [25].

Vincet et al. (2020) attempted to identify natural product molecules with potential inhibitory effects on COVID-19 through their interaction with the main protease (3CLpro). Molecular docking results indicated that around 15 compounds from Kabasura Kudineer (a poly-herbal formulation prescribed in AYUSH for COVID-19) plants, demonstrated superior energy scores compared to synthetic drugs, suggesting their potential as effective inhibitors of COVID-19. These findings warrant further testing and developing these compounds for potential use in antiviral drug development against coronaviruses [26].

Arisaema jacquemontii Blume, a plant from the Araceae family, is both highly medicinal and toxic, it has been traditionally used to treat various serious diseases, including viral infections, and exhibits properties such as antioxidants, anti-cancer, antimalarial, anti-vermicidal, and antiviral activities. The study conducted by Shehzadi et al. (2022) isolated 22 compounds from their methanolic extracts and conducted molecular docking against a selected COVID-19 protease protein (6LU7). Three compounds found in the rhizome demonstrated the most promising binding interactions against the SARS virus causing COVID-19, suggesting their potential to develop phytochemical-based COVID-19 therapeutics [27].

By investigating 198 bioactive compounds extracted from five specific medicinal plants known for their antiviral potential against SARS-CoV-2 protease and co-receptors, the study singles out Astragalin as a highly effective inhibitor for combating COVID-19. Astragalin exhibited exceptionally robust molecular interactions, both in terms of molecular docking binding energies and molecular dynamics simulations, suggesting its merit as a promising drug candidate for countering this virus [28].

Molecular docking analysis was conducted with 47 bioactive compounds derived from 10 commonly used Indian medicinal plants for treating cold and respiratory disorders, targeting key structures of SARS-CoV-2 (Mpro and spike protein) and the human ACE2 receptor. Through this analysis and subsequent molecular dynamics simulations, Cucurbitacin E and orientin were identified as potential candidates with high binding affinity, suggesting their potential as valuable scaffolds for the development of anti-COVID-19 drugs [29].

An interesting study focusing on the phytochemicals found in 38 medicinal plants from North-East India, known for their antiviral, antioxidant, or antibacterial properties assessed their drug-likeness. Initially, 231 phytochemicals underwent screening based on the Lipinski rule of five, resulting in 131 candidates. Further refinement using SWISS-ADME led to the selection of 50 phytochemicals. These compounds were subjected to molecular docking against the spike protein of the Delta variant (B.1.617.2) and the Delta-Plus (AY.1) variant of SARS-CoV-2, utilizing Autodock Vina and MOE 09. Among the 50 docked compounds, flavones demonstrated notably strong docking scores, particularly Poly-Methoxyflavones and Poly-Ethoxyflavones, which exhibited significant binding affinity to the spike protein. These findings suggest their potential as anti-viral drug candidates against the prevailing Delta variant of SARS-CoV-2 [30].

Over 16,500 compounds were screened against three viral targets (3CLpro, PLpro, and RdRp) identified constituents with the potential to inhibit SARS-CoV-2 activity by disrupting virus replication. Network pharmacology analysis highlighted compounds with multi-target properties and enriched pathways, underscoring the potential role of medicinal plants in managing SARS-CoV-2 infections, with a special focus on Glycyrrhiza glabra (liquorice), known for its historical use in treating viral infections. However, further validation through molecular dynamic simulations, in vitro, and in vivo studies is essential to substantiate these findings, offering valuable insights for the exploration and development of anti-SARS-CoV-2 therapeutic agents sourced from natural origins [31].

Despite the promising potential of ethnomedicine in treating various ailments, its effectiveness against SARS-CoV-2 remains an area for further exploration. Investigating the binding hotspots of key residues on potential candidates could uncover valuable opportunities for drug development. In addition, the broader application of these candidates and other plant-based ligands in treating viral infections beyond COVID-19, such as influenza and other respiratory diseases, warrants continued research. These insights highlight the practical significance of ethnomedicine in the evolving landscape of modern drug discovery.

## 3 Materials and Methods

### 3.1 Protein selection and preparation

The crystal structure of the SARS-CoV-2 spike protein (S) (PDB ID: 6VYB.pdb) was acquired from the RCSB Protein Data Bank [32]. This database contains experimentally identified and verified structures of macromolecules. The retrieved protein structure is pivotal as it facilitates the virus's entry into host cells and is a key trigger for antibody responses, making it a primary target for the proposed study. The protein structure was cleaned by removing unwanted chains utilizing UCSF Chimera (https://www.cgl.ucsf.edu/chimera/). Chimera is an open-source software that provides interactive visualization and analysis of molecular structures, such as density maps, supramolecular assemblies, sequence alignments, docking results, trajectories, and conformational ensembles. The protein structure was then prepared for docking using Chimera by performing energy minimization. The unmodelled regions in the protein structure were modeled using MODELLER v10.5 (https://salilab.org/modeller/). Subsequently, the prepared protein structure was saved for further docking analysis.

### 3.2 Ligand selection and preparation

The Sultanate of Oman has a wide variety of plant life, including some very special and rare medicinal plants. These plants are found in specific natural spots that have been formed by the country's unique desert landscapes. The process of selecting ligands began with a review of a plant database [33], which was compiled from an extensive list of medicinal plants documented by researchers in Oman [34]. Out of 437 medicinal plants identified across Oman, 47 species have been recognized and documented for their traditional use by indigenous tribes in treating respiratory infections, inflammations, and other diseases. From these, 30 species are cataloged in existing databases. The active ligands from these plants were downloaded using the ChEMBL database. ChEMBL is an open database that stores binding, functional, and ADMET information for a wide variety of drug-like bioactive compounds [https://www.ebi.ac.uk/chembl/]. The retrieval process resulted in 406 unique ligands after duplicate removal. Chimera was used to convert the *mol.2* ligand file into *pdb* format to prepare it for docking. The final ligand structure was saved in PDB format, compatible with the chosen docking software. The ligands used in this study are detailed in Supplemental online material 1. This comprehensive screening approach was relatively underexplored, introducing here geographic and cultural dimensions of exploring the pool of potential antiviral agents.

### 3.3 Domain analysis of sars-cov-2 spike ectodomain

The functional domain analysis of the protein was conducted using the InterPro database to retrieve the length and name of the protein domains. The InterPro is a public database (https://www.ebi.ac.uk/interpro/) encompassing protein families, domains, and functional sites. It uses predictive models, known as signatures, provided by multiple databases that create the InterPro consortium. These retrieved domains were visualized in PyMOL software (The PyMOL Molecular Graphics System, Version 3.0 Schrödinger, LLC).

### 3.4 Virtual screening of protein against ligand

The virtual screening of the protein SARS-CoV-2 Spike Ectodomain against the ligands was performed by GNINA (https://github.com/gnina/gnina). This molecular docking software uses convolutional neural networks (CNNs) for the scoring function. In addition, Autodock Vina was used which enabled to precisely assess the binding affinities of the target-ligand complexes and ranking them by the strength of their interactions. Compounds exhibiting the highest binding affinity were selected for further analysis.

### 3.5 Interaction analysis of protein-ligand complex

Interaction analysis was performed on the docked complex with the highest affinity score to highlight the interacting residues within the domain regions. Additionally, the 2D interactions of the protein-ligand complex were visualized using the DiscoveryStudio Visualizer (Biovia, D.S. (2019) Discovery Studio Visualizer. San Diego). This analysis unrevealed the potential formation of hydrogen bonds, and pi-pi interactions. Also, it provided a detailed understanding of the binding dynamics, guiding us toward a more refined model of protein-ligand interaction.

### 3.6 Molecular dynamic simulations of protein-ligand complex

The molecular dynamics (MD) simulation was conducted to observe the dynamic behavior of the protein-ligand complex by utilizing GROMACS 2023, a high-performance, most widely used open-source software for dynamic simulations of biomolecules [14].

The protein and ligand were preprocessed using chimera before the simulations were initiated. Initially, the ligand underwent preprocessing, which involved the incorporation of hydrogen atoms. This modification was executed utilizing the structural editing module of Chimera. Following the addition of hydrogen atoms, the pre-processed ligand was subsequently stored in an independent mol.2 file format. The protein preprocessing involved using the Dockprep tool of Chimera to remove unwanted molecules or ions from the structure. Hydrogens were added to generate protonation states, and partial charges were assigned to the atoms of standard and nonstandard residues. Subsequently, the preprocessed protein structure was saved in a separate PDB file.

The protein and ligand topologies were generated independently after preprocessing. Initially, the topology of the protein was constructed utilizing the pdb2gmx module. The CHARMM27 force field and the TIP3P water model were employed to generate the protein topology. Following the generation of the protein topology, the topology of the ligand was also generated. Generating a ligand topology is the most crucial step in MD simulations of a complex. It is also difficult to deal with bound ligands in MD simulations. Therefore, the automated program SwissParam (http://www.swissparam.ch/) was employed to create the ligand topology. The next step involved creating the topology of the entire complex. After the complex topology was generated, it was placed inside a box and solvated using the solvate module. Ions were then added to neutralize the system with the genion module. Before running MD, the ions and the solvent were equilibrated around the protein. Subsequently, the energy of the entire system was minimized to stabilize the structure and eliminate any steric clashes. The energy minimization process involved two equilibration phases. The initial phase, known as NVT (constant Number of atoms, Volume, and Temperature), ran for 100 ps using an ensemble with a constant temperature of 300 K and a coupling constant of 0.1 ps. The second phase of NPT (constant number of atoms, pressure, and temperature) ran for 100 ps using an ensemble with constant pressure of 1 bar and was employed with a coupling constant of 5 ps. The final production MD run of 50 ns was performed for the complex to investigate the structural stability and dynamics.

### 3.7 Analysis of MD simulations trajectories

MD simulation results were analyzed using the. xtc trajectory file generated after the simulations to observe the structural stability of the complex. Post-simulation analysis was performed, including the root mean square deviation (RMSD), root mean square fluctuations (RMSF), and hydrogen bond analysis. These analyses were carried out using commands like **gmx rms, gmx rmsf, and gmx hbond,** respectively. Moreover, three parameters were examined to assess ligand properties, such as Solvent Accessible Surface area (SASA), Polar Surface Area (PSA), and Radius of Gyration (rGyr).

The in-depth analysis of trajectories derived from Molecular Dynamics (MD) simulations was conducted utilizing Essential Dynamics (ED) or Principal Component Analysis (PCA). PCA facilitated the identification of protein motion by extracting the prominent motion patterns of the protein across various frames throughout the simulations. PCA was performed through a Python package named MDTraj using the GROMACS trajectory files.

## 4 In-vitro approach

### 4.1 Inhibitory assay for sars-cov-2 spike protein

The assay employed to measure the interaction between the viral Spike protein's Receptor Binding Domain (RBD) and the hACE2 cell surface utilized the BPS Bioscience/Tebu-bio kit from Offenbach, Germany [35]. This process involved a colorimetric assay to investigate the binding affinity of the spike S1 RBD with Mouse Fc-fusion (SARS-CoV-2) and to identify potential antiviral compounds [36]. Initially, ACE2 was coated onto a 96-well plate at 50 ng/well, followed by the addition of 100 ng/well of RBD/S1 protein to facilitate interaction with the ACE2 [37]. The assay aimed to determine if the S-protein complex could adhere to ACE2 using the streptavidin-horseradish peroxidase (HRP) substrate in a setup involving a 96-well plate, where each well received 50 μL of ACE2 and was agitated gently for an hour at 25 °C. After incubation, the plate was washed three times with a 100 μL/well washing buffer. Subsequently, 100 μL of blocking buffer was applied to each well, incubated for 10 min, and then washed. Various concentrations of Corilagin (0.5, 0.25, 0.125, 0.0625, 0.0312, 0.0156, 0.0078, 0.0039, 0.0019 mM) were added at 30 μL/well. The assay proceeded with an hour of incubation at room temperature, after which 50 μL/well of blocking buffer 2 was used as a blank and 30 μL/well as a positive control. Following this, Spike S1 (0.0625 ng/L) at 20 μL/well was introduced to both test and positive control wells. The addition of 100 μL/well of HRP-labelled antibody (anti-mouse) in blocking solution 2 preceded a room-temperature incubation with gentle shaking. The reaction was developed using 100 μL/well of colorimetric HRP substrate until the positive control showed a blue coloration, after which 100 μL/well of 1N HCl was added to stop the reaction, and readings were taken at 450 nm.

### 4.2 Statistical analysis

The programs were employed to analyze the attained results for biological activity, the SoftMax Pro package and Excel were utilized. The formula used to calculate percent inhibition is shown in Equation (1).

(1) $$\begin{array}{c} \%\text{Inhibition}=100‐\left(\frac{{O.D}_{\text{test}\;\text{compound}}}{{O.D}_{\text{control}}}\right)\times 100 \end{array}$$

EZ-FIT (Perrella Scientiﬁc, Inc., USA) was used for IC_50_ calculations of all tested samples. To overcome the expected errors, all experiments were performed in triplicate, and variations in the results are reported in Standard Error of Mean values (SEM) (Equation (2)).

(2) $$\begin{array}{c} SE=\frac{\sigma }{\sqrt{n}} \end{array}$$

## 5 Results

### 5.1 Structure retrieval and virtual screening

**Fig. 1:**
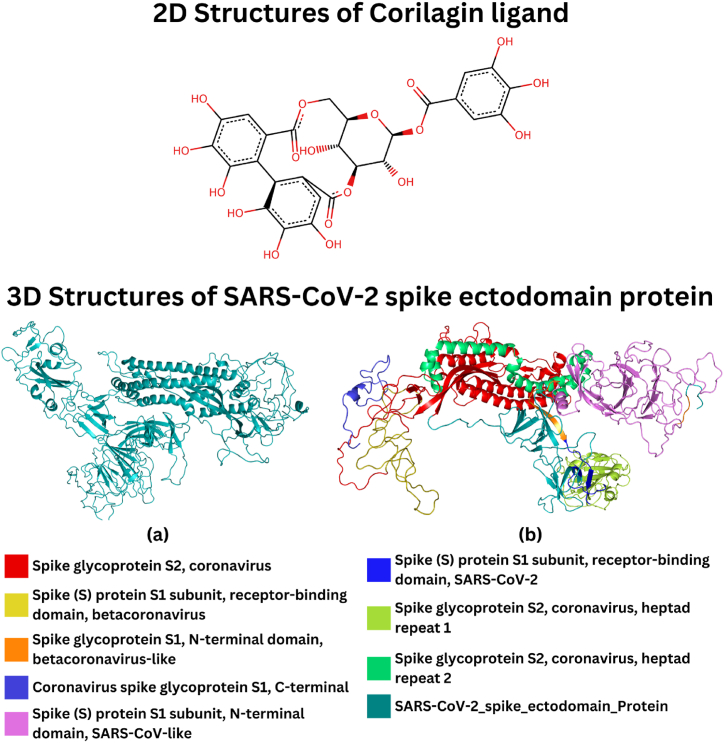
The 2D illustration of ligand Corilagin **(a)** Overview representation of Protein structure and domain analysis, **(b)** a 3D representation of 8 different domains of the protein shown in different colors.

**Fig. 2:**
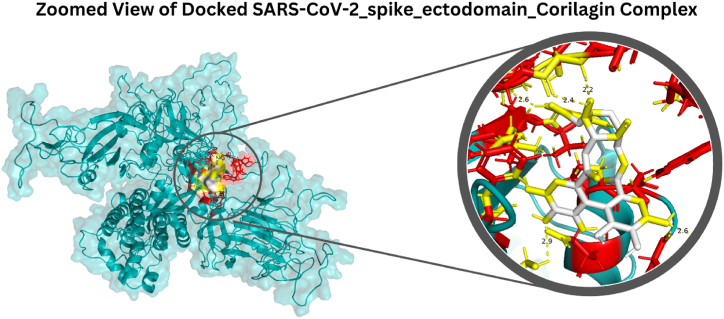
The zoomed view illustration of the protein structure of protein screened against Corilagin.

The 3D structure of the SARS-CoV-2 spike ectodomain protein, with 1281 amino acids sequence length, was retrieved and prepared (cleaned, minimized, and modeled). Moreover, eight domains were identified for this protein (Table 1). Among 406 screened ligands, the top ten ligands showing the highest binding affinities are detailed in Table 2. The structure of the highest binding affinity (Corilagin = −11.36 kcal/mol) was retrieved and converted into the *pdb* format. The 3D structure of protein and the 2D structures of ligand are represented in Fig. 1(a and b). The zoomed view of the screened complex is illustrated in Fig. 2. Corilagin is a tannin commonly found in various parts of the *Acalypha indica* plant, including the leaves, stems, and roots [38]. The concentration of Corilagin varies depending on the plant part, environmental factors, and extraction methods. Studies have reported that Acalypha indica contains various phytochemicals and nutrients, but it also has toxic effects, such as poisoning and methemoglobinemia, due to compounds like quinine and 2-methyl anthraquinone. Despite this, Corilagin stands out due to its demonstrated pharmacological potential, particularly its antiviral, anti-inflammatory, and antioxidant properties. These characteristics make it a valuable candidate for further investigation. While natural mixtures may sometimes enhance therapeutic effects, focusing on single components like Corilagin allows for a detailed understanding of their specific interactions with biological targets, such as the SARS-CoV-2 spike protein. This approach could inform the development of targeted therapies and optimize the compound's potential use in combination with other treatments.

**Table 1:** Overview of protein domains and their corresponding regions.

Protein	Domains	Region
SARS-CoV-2 spike ectodomain	Spike glycoprotein S2, coronavirus	711–1232
	Spike (S) protein S1 subunit, receptor-binding domain, betacoronavirus	334–527
	Spike glycoprotein S1, N-terminal domain, betacoronavirus-like	9–337
	Coronavirus spike glycoprotein S1, C-terminal	536–592
	Spike (S) protein S1 subunit, N-terminal domain, SARS-CoV-like	13–304
	Spike (S) protein S1 subunit, receptor-binding domain, SARS-CoV-2	319–541
	Spike glycoprotein S2, coronavirus, heptad repeat 1	896–1001
	Spike glycoprotein S2, coronavirus, heptad repeat 2	1143–1225

**Table 2:** Top ten ligands associated with the highest binding affinity.

Family	Plant Name	Local Name	Plant Part	Traditional use	Phytochemical name of ligand	References	Binding Affinity
Euphorbiaceae	Acalypha indica	Aeyan Al Aqrada	Whole plant	Used for asthma	Corilagin	ISBN:9770976605004	−11.36 kcal/mol
Apocynaceae	Nerium oleander	Haban	Leaf	Leaves are used to cure sinusitis and bronchitis	Adynerigenin beta-neritrioside	ISBN:9788185042145	−10.6 kcal/mol
Apocynaceae	Nerium oleander	Haban	Leaf	Leaves are used to cure sinusitis and bronchitis	Isoneriucoumaric acid	ISBN:9788185042138	−10.3 kcal/mol
Amaranthaceae	Celosia argentea	Al-Deek	Flower	Anti-inflammatory and disinfectant	Celosianin II	ISBN:9780387706375	−10 kcal/mol
Solanaceae	Datura metel	Meranha	Flower and leaf	Flowers and leaves smoked as a treatment for asthma	withametelin	ISBN:9770972795006, ISBN:9788172362300, ISBN:9788185042053	−10 kcal/mol
Asphodelaceae	Asphodelus tenuifolius	kawther	Whole plant	Treat cold	Asphodelin	ISBN:9788172362089	−10 kcal/mol
Solanaceae	Datura metel	Meranha	flower and leaf	Flowers and leaves smoked as a treatment for asthma	Isowithametelin	ISBN:9770972795006, ISBN:9788172362300, ISBN:9788185042138	−10 kcal/mol
Apocynaceae	Nerium oleander	Haban	Leaf	Leaves are used to cure sinusitis and bronchitis	Odoroside K	ISBN:9788185042053	−10 kcal/mol
Apocynaceae	Nerium oleander	Haban	Leaf	Leaves are used to cure sinusitis and bronchitis	Adynerigenin beta-odorotrioside	ISBN:9788185042145	−10 kcal/mol
Euphorbiaceae	Euphorbia hirta	Eshbat alrabui	Whole plant	Used in asthma and bronchitis	xanthorhamnin	ISBN:9788172361266	−9.9 kcal/mol

### 5.2 Interaction analysis of screened protein-ligand complex

**Fig. 3:**
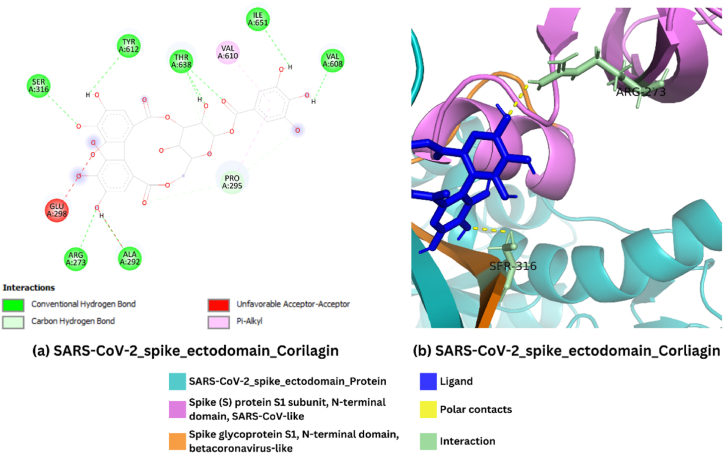
Interaction analysis of the docked complex **(a)** 2D representation of the interaction of the protein with the ligand **(b)** 3D representation of protein-ligand interactions.

The interaction analysis was performed on the protein-ligand complex to highlight the interactions and identify the interacting residues between the protein and ligand. The SARS-CoV-2 spike ectodomain protein exhibited multiple interactions with corilagin at residues ARG (273), (ALA 292), GLU (298), and PRO (295) within two domains (Spike (S) protein S1 subunit, N-terminal domain, SARS-CoV-like domain (13–304) and Spike glycoprotein S1, N-terminal domain, betacoronavirus-like (9–337)). Similarly, the protein exhibited interaction at residue SER (316) within the Spike glycoprotein S1, N-terminal domain, betacoronavirus-like domain (9–337). However, the interactions at residues VAL (608), VAL (610) TYR (612), THR (638), and ILE (651) occurred outside the domain regions. The 2D and 3D protein-ligand interactions are illustrated in Fig. 3. These binding hotspots on spike protein where Corilagin interacts, including key residues involved in viral entry. This mapping deepens the Corilagin mechanism to inhibit viral infection and might be considered valuable insights for future drug design efforts. Optimizing these interactions may lead to the development of even more potent derivatives.

### 5.3 Molecular dynamic simulations of docked complex

**Fig. 4:**
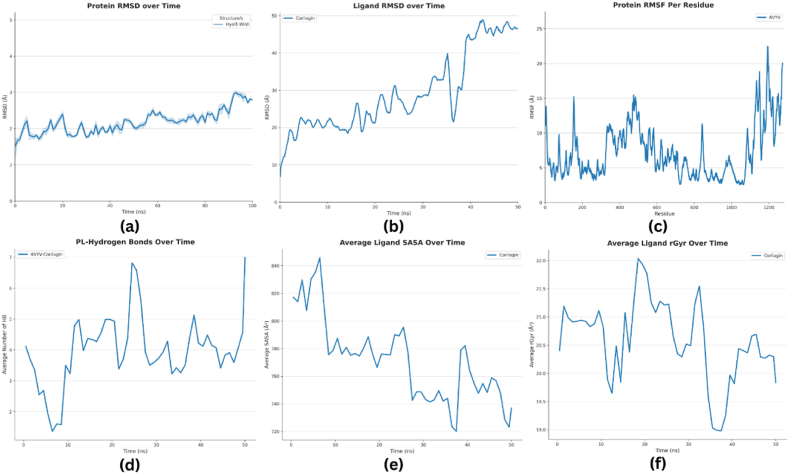
Visualization of post-simulation analysis **(a)** RMSD of protein over the course of time **(b)** RMSD of ligand over the course of time **(c)** MD simulation illustration depicting the RMSF per residue for protein in complex with ligand **(d)** MD simulation results depicting the average hydrogen bond numbers **(e**–**f)** Ligand properties **(e)** Solvent available surface area **(f)** Radius of gyration.

The MD simulations for the protein-ligand complex were conducted to observe the structure stability and flexibility. The RMSD plot revealed that the protein attained an equilibrium state from approximately 40 ns–45 ns during the simulation. The RMSD value of the protein ranged from the lowest value of 4.2 Å to the highest value of 20.3 Å, as illustrated in Fig. 4 (a). The high RMSD values of the protein implicated that the complex was highly unstable. The RMSD plot of the ligand revealed that the ligand was highly unstable with the RMSD value of 50.7 Å at the end of the simulation time, as represented in Fig. 4 (b). The protein RMSF values showed that the protein residues fluctuated significantly; however, the protein residue ASN at position 1192 showed the highest fluctuations with the RMSF values of 22 Å, as shown in Fig. 4 (c).

Moreover, the hydrogen bond analysis revealed the average number of hydrogen bonds between protein and ligand throughout the simulation. The number of hydrogen bonds between protein and ligand showed high fluctuations, suggesting the dynamic interactions between the protein and the ligand. Furthermore, the highest number of hydrogen bonds were formed at 25 ns and 50 ns, suggesting that these time points might be significant in the interaction dynamics between the protein and the ligand. These time points could represent specific conformational states where the protein and ligand are optimally positioned to form hydrogen bonds. The average number of hydrogen bonds plotted against simulation time (ns) is illustrated in Fig. 4 (d).

Ligand properties were assessed by analyzing SASA and Radius of Gyration plots. SASA is defined as a parameter for the surface area of protein mainly assessable to solvents. Based on the SASA plot, the ligand showed a value of 90 nm^2^, as depicted in Fig. 4 (e). However, the value of SASA significantly decreased, indicating that the complex had become more compact or less extended by the end of the simulation making the complex highly unstable. Lastly, the radius of the gyration plot showed a lower value of 4.9 nm, indicating that the molecule is more extended or less compact, implying lower stability due to increased flexibility (Fig. 4 (h)).

**Fig. 5:**
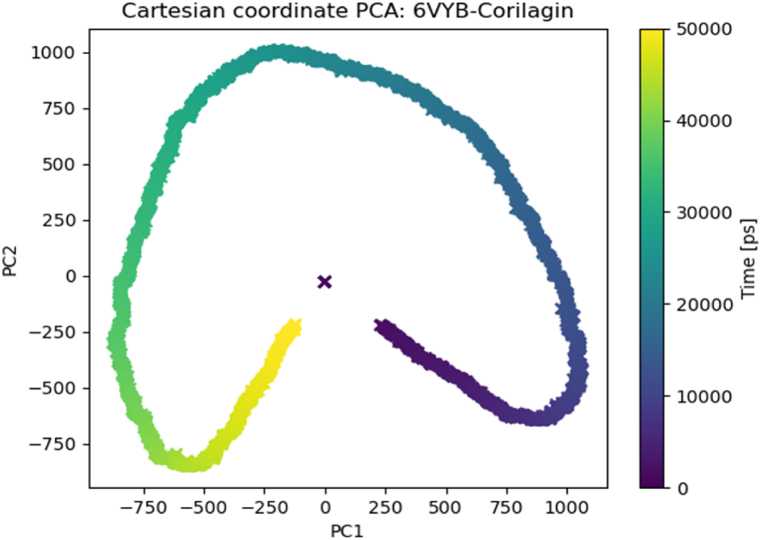
Principal component analysis Plot. The X-axes represent the principal component 1, and the Y-axes represent PC2, which captured the most significant modes of motion in the system.

Essential dynamics or PCA was used for a broader view of dynamic properties for MD simulation results. The projection of the first two principal components in phase space for the complex explained the cluster of the complex was expanded in the conformational space due to flexibility Moreover, the elliptical or loop-like shape of the plot suggested collective motions within the protein-ligand complex. Such motions could involve domain movements, ligand binding/unbinding, or conformational changes. The projection of the first two principal components in reduced dimensional space is illustrated in Fig. 5.

### 5.4 IN-VITRO inhibitory expirment

**Fig. 6:**
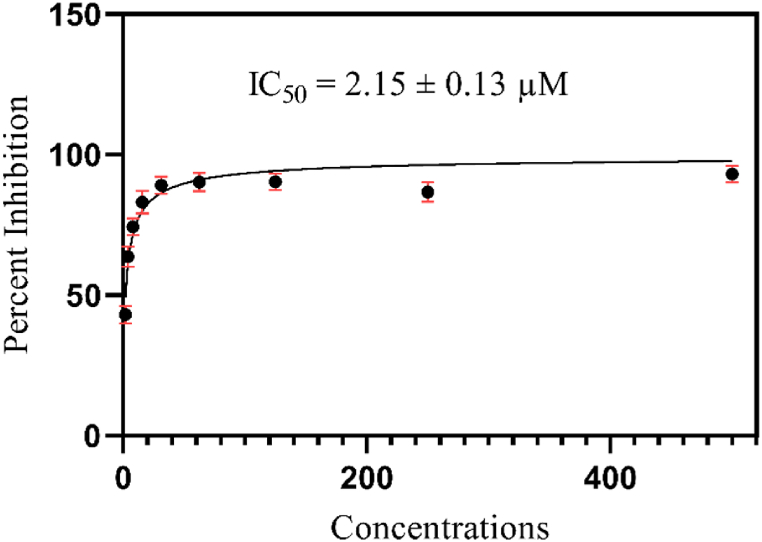
Dose curve response for Corilagin inhibitory activity against SARS-CoV-2.

While many computational studies remain in-silico, this study takes a step further by empirically verifying Corilagin's inhibitory actions using in-vitro experiments. This combined method increases the credibility of Corilagin's medicinal potential. The in-vitro inhibitory effect of the compound Corilagin was evaluated against SARS-CoV-2 to insight into their therapeutic potential. Corilagin exhibited 92 % inhibition at 0.5 mM. Additionally, further investigations were conducted to determine the lowest concentration of Corilagin that inhibits 50 % of its activity, aiming for an accurate IC_50_ calculation. The testing was carried out with various concentrations of Corilagin, including 0.5, 0.25, 0.125, 0.0625, 0.0312, 0.0156, 0.0078, 0.0039, and 0.0019 mM. Fortunately, this compound exhibited higher potency at micromolar concentration (IC_50_ = 2.15 ± 0.13 μM) (Fig. 6). These significant findings confirm its computational predictions and also demonstrate its strong potential for drug development in a biological setting. However, this must be considered as an initial step in evaluating the potential antiviral effects of Corilagin. Several studies have recently identified that natural products, and their derivatives possess significant inhibitory potential for SARS-CoV-2 spike protein [[39], [40], [41], [42]]. Those studies further correlate well with our findings that Corilagin as a natural product holds a significant position as a drug-like candidate for SARS-CoV-2.

## 6 Conclusion

Our study provides a comprehensive evaluation of Corilagin, a phytochemical derived from *Acalypha indica*, locally referred to as Aeyan Al Aqrada, as a potential therapeutic for COVID-19. By screening a large number of ligands from traditionally used Omani medicinal plants, we introduce a novel geographic and ethnopharmacological perspective into modern drug discovery. The integration of in-silico and in-vitro methodologies strengthens the validity of our findings, demonstrating the high inhibitory potential of Corilagin, as evidenced by a 92 % inhibition rate and an IC_50_ value of 2.15 μM. These findings, combined with the detailed interaction mapping of key viral residues, pave the way for further optimization of Corilagin derivatives and offer promising new avenues for antiviral drug development. We recommend further studies to explore its pharmacokinetics, bioavailability, and toxicity profiles. Additionally, structural optimization of Corilagin, along with advanced in-vivo testing, will be necessary to move closer to clinical trials. We also encourage broader exploration of the therapeutic potential of other ligands identified from Omani medicinal plants, which may yield additional valuable candidates for antiviral drug development.

## CRediT authorship contribution statement

**Nabras Al-Mahrami:** Formal analysis, Data curation. **Smitha Sunil Kumaran Nair:** Writing – review & editing, Writing – original draft, Funding acquisition, Data curation, Conceptualization. **Adhra Al Mawali:** Writing – review & editing, Project administration. **Raja-Mohamed Beema Shafreen:** Writing – review & editing, Conceptualization. **Saeed Ullah:** Validation, Investigation. **Sobia Ahsan Halim:** Writing – review & editing, Supervision. **Ahmed Al-Harrasi:** Supervision, Project administration. **Nallusamy Sivakumar:** Writing – review & editing, Resources.

## Disclosure statement

The authors report there are no competing interests to declare.

## Declaration of competing interest

All the listed authors have read and approved the submitted manuscript.

This manuscript/data, or parts thereof, has not been submitted for possible publication to another journal or that the work has previously been published elsewhere.

The present study was performed according to international, national and institutional rules considering animal experiments, clinical studies and biodiversity rights.
